# Phenotypic Analysis of Embryos in a Noonan Syndrome Model Mouse With the *Rit1* A57G Mutation

**DOI:** 10.1002/mgg3.70167

**Published:** 2025-12-13

**Authors:** Dai Suzuki, Taiki Abe, Tetsuya Niihori, Atsuo Kikuchi, Yoko Aoki

**Affiliations:** ^1^ Department of Medical Genetics Tohoku University School of Medicine Sendai Japan; ^2^ Department of Pediatrics Tohoku University School of Medicine Sendai Japan

**Keywords:** hypertrophic cardiomyopathy, lymphatic vessel, MEK1/2 inhibitor, Noonan syndrome, Rit1A57G

## Abstract

**Background:**

Noonan syndrome is a congenital genetic disorder characterized by distinctive craniofacial features, short stature, and congenital heart disease. Dysregulation of the RAS/mitogen‐activated protein kinase (MAPK) pathway is a common molecular mechanism underlying the pathogenesis of these disorders. Germline mutations in *RIT1* have also been identified in patients with Noonan syndrome. Patients with *RIT1* mutations frequently exhibit cardiovascular abnormalities such as hypertrophic cardiomyopathy and lymphatic disorders. However, it remains unclear when cardiovascular abnormalities and lymphatic disorders develop and whether these disorders influence prognosis during the fetal period.

**Methods:**

We investigated the cardiovascular and lymphatic phenotypes of *Rit1* ^A57G/+^ embryos. To elucidate that the activation of MEK/ERK is the involvement of cardiac abnormalities in *Rit1* ^A57G/+^ embryos, we administered a MEK1/2 inhibitor to *Rit1* ^A57G/+^ embryos and investigated the cardiovascular phenotypes.

**Results:**

At E16.5, *Rit1* ^A57G/+^ embryos exhibited cardiac hypertrophy without cardiomyocyte hypertrophy and demonstrated progressive cell proliferation. Furthermore, *Rit1* ^A57G/+^ embryos exhibited pulmonary valve stenosis and lymphatic vessel expansion. Maternal intraperitoneal injection of PD0325901, a MEK1/2 inhibitor, prevented cardiac hypertrophy in *Rit1* ^A57G/+^ embryos.

**Conclusions:**

*Rit1* mutation causes cardiovascular and lymphatic abnormalities in the fetal period, and that the activation of MEK/ERK is the potential pathogenesis of cardiac hypertrophy.

## Graphical abstract

*Rit1* ^A57G/+^ embryos exhibited cardiac hypertrophy and lymphatic vessel dilatation. The administration of the MEK1/2 inhibitor ameliorated LV myocardial wall hypertrophy. The MEK/ERK activation would be one of the pathogenetic mechanisms of cardiac hypertrophy of *Rit1* ^A57G/+^ embryos.

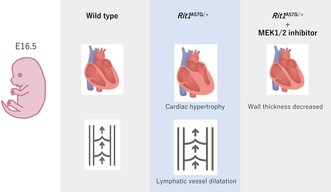

## 1 Introduction

Noonan syndrome (NS; OMIM 163950) is an autosomal dominant or rarely autosomal recessive congenital anomaly. NS is characterized by several clinical features, including distinctive facial appearance, congenital heart diseases, short stature, skeletal abnormalities, variable cognitive impairment, cryptorchidism in males, and lymphatic malformations. The common facial features are hypertelorism, downslanting palpebral fissures, and low‐set and posteriorly rotated ears. This syndrome is marked by considerable phenotypic variability, which is driven by underlying genetic heterogeneity. Regarding congenital heart disease, pulmonary valve stenosis and hypertrophic cardiomyopathy are common in NS (Aoki et al. 2016; Roberts et al. 2013; Saint‐Laurent et al. 2023). The estimated prevalence of this syndrome is between 1 in 1000 and 1 in 2500 live births (van der Burgt 2007).

The RAS/mitogen‐activated protein kinase (MAPK) pathway is a signal transduction pathway that is well‐studied and widely significant. It involves exogenous ligands such as growth factors, cytokines, and hormones for promoting cell proliferation, differentiation, survival, and metabolism. These extracellular stimulations activate the RAS/MAPK signaling pathway by phosphorylating tyrosine kinase receptors on the plasma membrane and convert RAS, a small GTPase, from the GDP‐bound inactive form to the GTP‐bound active form, which phosphorylates downstream RAF, stimulating the downstream MEK/ERK (Aoki et al. 2016; Roberts et al. 2013). RAS also activates other downstream effectors, including phosphatidylinositol 3 kinase (PI3K), phospholipase C, and RAL guanine nucleotide exchange factor (Tajan et al. 2018). Many studies have demonstrated that activation of the RAS/MAPK pathway is associated with phenotypically similar genetic disorders, known as RASopathies (Aoki et al. 2008). RASopathies include several syndromes such as NS, cardio‐facio‐cutaneous syndrome (OMIM 115150), Costello syndrome (OMIM 218040), and Noonan syndrome with multiple lentigines (NSML; OMIM 151100). NS is a common type of RASopathy. Missense mutations in *protein‐tyrosine phosphatase, non‐receptor type 11* (*PTPN11*), which encodes tyrosine phosphatase Src homology region 2‐containing protein tyrosine phosphatase 2 (SHP‐2), were identified as the first causative gene of NS (Tartaglia et al. 2001). The primary causative genes and their respective frequencies in NS have been identified as follows: *PTPN11* (40.9%) (Romano et al. 2010), *Son of sevenless homolog 1* (*SOS1*, 19.3%) (Tartaglia et al. 2007), *Raf‐1 proto‐oncogene* (*RAF1*, 10.2%) (Pandit et al. 2007), and *Ras‐like without CAAX1* (*RIT1*, 9%) (Aoki et al. 2013).

In 2013, we identified germline *RIT1* (OMIM 609591) mutations in patients with NS using Sanger and exome sequencing (Aoki et al. 2013). RIT1 is a member of the Ras subfamily that directs broad cellular physiological responses through tightly controlled signaling networks. A distinctive feature of the hypervariable region in RIT1 is the absence of a CAAX box motif that permits anchoring of RIT1 to the plasma membrane via lipid moieties (Van et al. 2020). *RIT1* is located at the 1q22 locus and spans a genomic length of 13.5 kb, comprising six exons and five introns. In humans, RIT1 is expressed in many tissues, particularly the lungs, esophagus, blood, vagina, and spleen (Consortium 2013). Somatic mutations in this gene have been reported to cause lung adenocarcinoma and arteriovenous malformations (Berger et al. 2014; Kapp et al. 2024).

Cardiac and lymphatic abnormalities are more common in NS cases caused by *RIT1* mutations than in NS cases caused by other genetic mutations (Kouz et al. 2016; Zha et al. 2022). We previously reported that all patients with NS with *RIT1* mutations had cardiovascular abnormalities (Yaoita et al. 2016). All patients had lymphatic disorders such as polyhydramnios and chylothorax during the prenatal or neonatal period. In a previous study, we generated a novel NS mouse model with a *Rit1* p.A57G mutation and demonstrated that *Rit1* ^A57G/+^ adult mice exhibited hypertrophic cardiomyopathy (HCM) with progressive cell proliferation and fibrosis (Takahara et al. 2019). Other groups have reported that patients with NS harboring an *RIT1* mutation frequently exhibit prenatal or neonatal abnormalities associated with cardiovascular and lymphatic disorders (Kouz et al. 2016; Zha et al. 2022). However, the precise timing of the onset of cardiovascular and lymphatic abnormalities and their potential impact on fetal prognosis remains unclear.

In the present study, we investigated the cardiovascular and lymphatic phenotypes of *Rit1* ^A57G/+^ embryos and evaluated whether a MEK1/2 inhibitor prevents cardiovascular defects in *Rit1* ^A57G^ embryos.

## 2 Materials and Methods

### 2.1 Mice

Heterozygous knock‐in mice expressing the *Rit1* ^A57G^ mutation have been previously reported (Takahara et al. 2019). Male *Rit1* ^A57G/+^ mutant and female wild‐type mice were mated for 12 h and subsequently isolated. Fourteen or 16 days after isolation, we euthanized pregnant mice and analyzed the embryos at embryonic days (E)14.5 and E16.5. The mice were maintained under a 12‐h light/12‐h dark cycle. Animal experiments were approved by the Animal Care and Use Committee of Tohoku University and performed in accordance with the guidelines for animal experimentation at Tohoku University (Approval No. 2020MdA‐095‐01, 2019MdLMO‐171).

### 2.2 Genotyping

Genomic DNA was extracted from the tail tissues. DNA purification was performed as previously described (Abe et al. 2023, 2024). Genotyping of *Rit1* ^+/+^ and *Rit1* ^A57G/+^ mice was performed by polymerase chain reaction (PCR) using KOD FX Neo (TOYOBO, Osaka, Japan). The primers used for PCR have been described previously (Takahara et al. 2019).

### 2.3 Histology and Immunohistochemistry

Histological and immunohistochemical sections were prepared as previously described (Inoue et al. 2017; Takahara et al. 2019). The sections were stained with hematoxylin (Muto Pure Chemicals, Tokyo, Japan) and eosin (Wako, Osaka, Japan) (H&E) and a TUNEL staining kit (MK500, Takara Bio, Japan), according to the manufacturer's protocols. Immunohistochemistry was performed using a Histofine Simple Stain Kit (Nichirei Bio Sciences, Tokyo, Japan) and antibodies against VEGFR3/Flt4 (AF74; R&D Systems, Minneapolis, MN, USA) and Ki‐67 (418,071; Nichirei Bio Sciences). Subsequently, signals were visualized using a DAB Substrate Kit (Nichirei Bio Sciences). The number of identifiable cardiomyocytes was counted in two randomly selected fields on the left ventricular myocardial wall using the ImageJ Fiji software. The selected areas were divided by the number of cardiomyocytes to determine the cardiomyocyte size.

### 2.4 MEK1/2 Inhibitor Administration for Mice

Mirdametinib (PD0325901, 168‐25293; Wako), a MEK1/2 inhibitor, was resuspended in dimethylsulfoxide and then diluted 50‐fold in corn oil to a final concentration of 0.1 mg/mL. Mirdametinib (1.0 mg/kg body weight) was injected intraperitoneally into pregnant mice on gestational day 12.5 and 14.5. The mice were euthanized on gestational day 16.5, and the embryos were analyzed. The administration methods were described previously (Inoue et al. 2014).

### 2.5 Statistical Analyses

Statistical analyses were performed using JMP Pro17 software (SAS, Cary, NC, USA). Data are presented as the interquartile range (IQR, median). Significant differences between two groups were assessed using the Wilcoxon signed‐rank sum test or Fisher's exact test, as applicable. Differences were considered statistically significant at *p* < 0.05.

## 3 Results

### 3.1 Embryonic Phenotypes of *Rit1* ^A57G^ ^/+^ Mice

**FIGURE 1:**
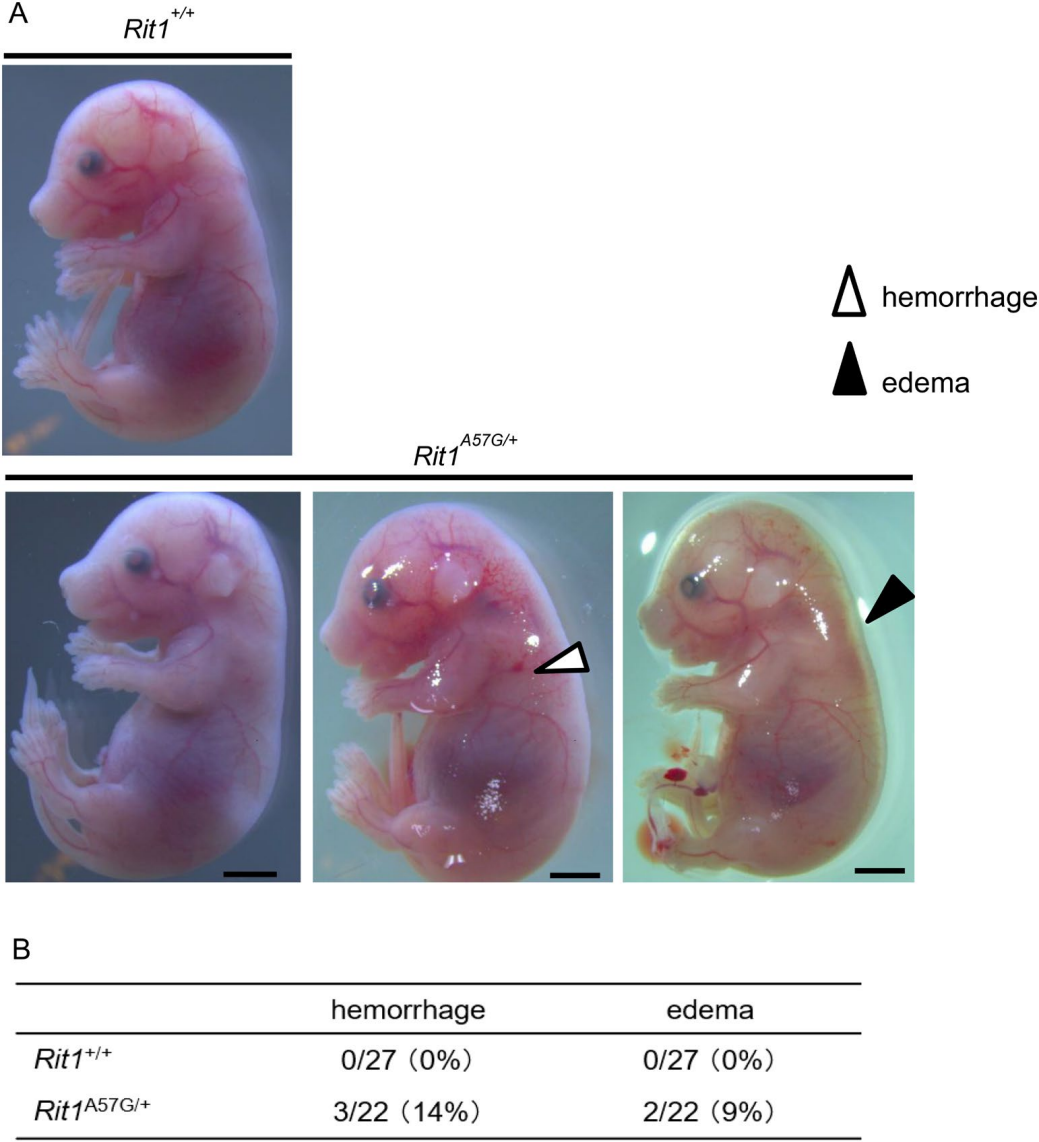
Gross appearance of Rit1^A57G/^+ and Rit1^+/+^ embryos at E16.5. (A) Representative images of Rit1^A57G/+^ and Rit1^+/+^ embryos at E16.5. White and black arrowheads indicate the subcutaneous hemorrhage and edema, respectively. Scale bars: 2 mm. (B) The number and ratio of subcutaneous hemorrhages and edema in Rit1^A57G/^+ and Rit1^+/+^ embryos at E16.5.

To identify the genotype of embryos from the intercross between male *Rit1* ^A57G/+^ and female *Rit1* ^+/+^ mice, we performed PCR on the genomic DNA extracted from the tail tip. At E14.5 and E16.5, the ratio of *Rit1* ^A57G/+^ to *Rit1* ^+/+^ embryos was similar (Table 1). Conversely, the ratio of *Rit1* ^A57G/+^ in E18.5 embryos and P1 neonates showed a decreasing trend. Subsequently, we investigated the phenotypes of the E16.5 mice. A few *Rit1* ^A57G/+^ embryos showed subcutaneous hemorrhage and edema, whereas *Rit1* ^+/+^ embryos showed no observable abnormalities (Figure 1A,B). These results suggest that *Rit1* ^A57G/+^ embryos exhibit embryonic lethality after E16.5.

**TABLE 1:** Genotyping of pups resulting from intercross between male *Rit1* ^A57G/+^ and female *Rit1* ^+/+^ mice.

	Number of *Rit1* ^+/+^ embryos (%)	Number of *Rit1* ^A57G/+^ embryos (%)	Total number of embryos
E14.5	23 (51)	22 (49)	45
E16.5	44 (55)	36 (45)	80
E18.5	5 (71)	2 (29)	7
P1	18 (64)	10 (36)	28

### 3.2 *Rit1* ^A57G^ ^/+^ Embryos Exhibited Lymphatic Abnormalities

To investigate the relationship between embryonic lethality and lymphatic abnormalities in *Rit1* ^A57G/+^ embryos, we performed VEGFR3 immunostaining at E16.5 and measured three cross‐sectional areas of lymphatic vessels in the horizontal section. In *Rit1* ^A57G/+^ embryos, lymphatic vessels located in the head, mandible, and neck exhibited hyperplasia compared to those in wild‐type embryos (wild‐type *n* = 7, *Rit1* ^A57G/+^ *n* = 8) (Figure S1A–C).

### 3.3 *Rit1* ^A57G^ ^/+^ Embryos Exhibited Various Cardiovascular Abnormalities

**FIGURE 2:**
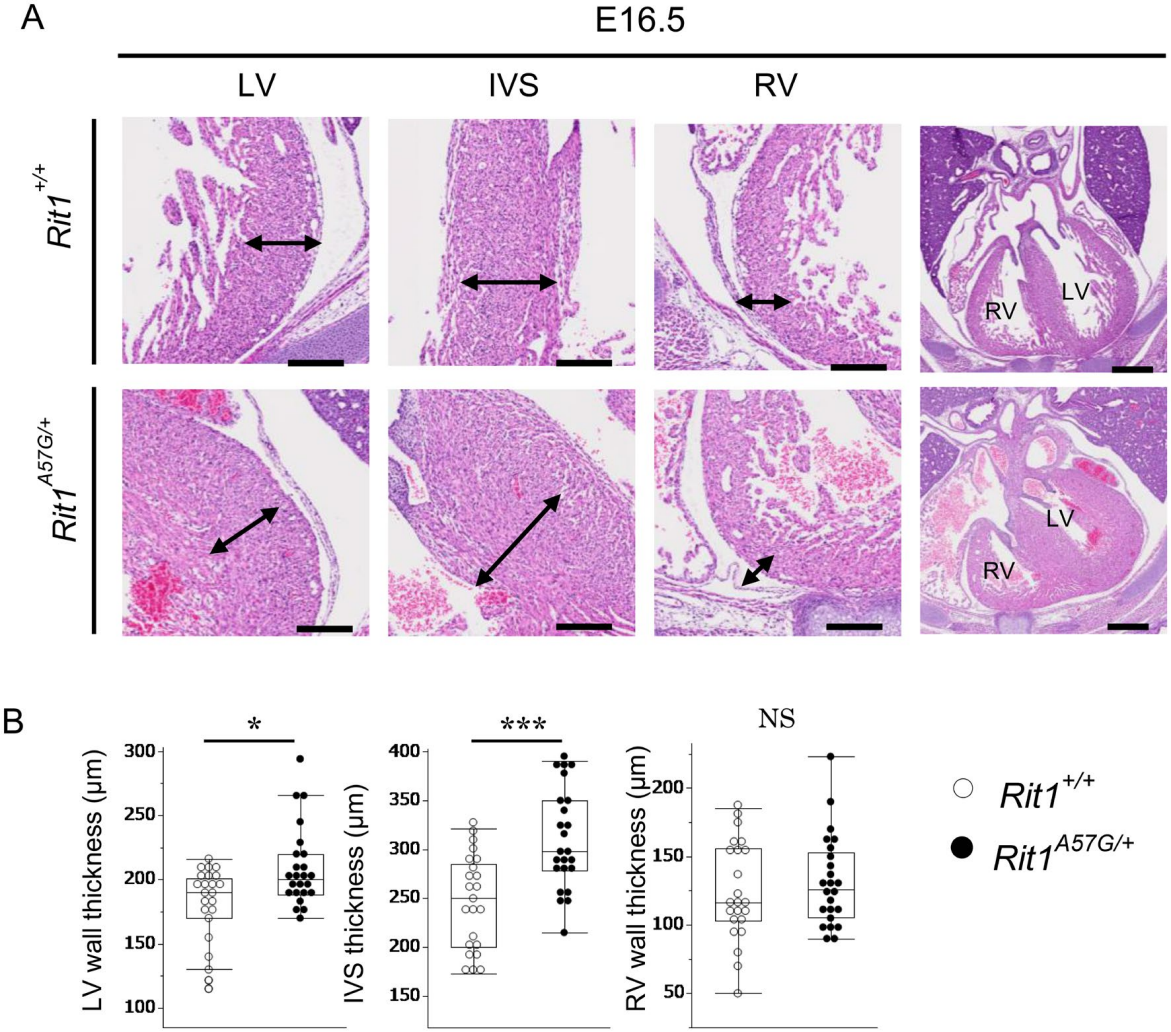
Rit1^A57G/+^ embryos exhibited ventricular hypertrophy at E16.5. (A) Representative images of heart sections stained with hematoxylin and eosin at E16.5. The upper panels show Rit1^+/+^ embryos, and the lower panels show Rit1^A57G/+^ embryos. High magnification images indicate the LV wall, IVS, and RV wall of the heart, and low magnification images show a four‐chamber view of the heart. The measured wall thickness is shown for each high‐magnification image. LV, left ventricle; IVS, interventricular septum; RV, right ventricle; Scale bars, 500 μm (low‐magnification) and 200 μm (high‐magnification). (B) Ventricular wall thickness was measured and compared between Rit1^+/+^ and Rit1^A57G/+^ embryos. Data are presented as median/IQR, Rit1^+/+^ (*n* = 23), Rit1^A57G/+^ (*n* = 24), *p < 0.05, ***p < 0.001, NS, not significant (Wilcoxon signed‐rank sum test).

**FIGURE 3:**
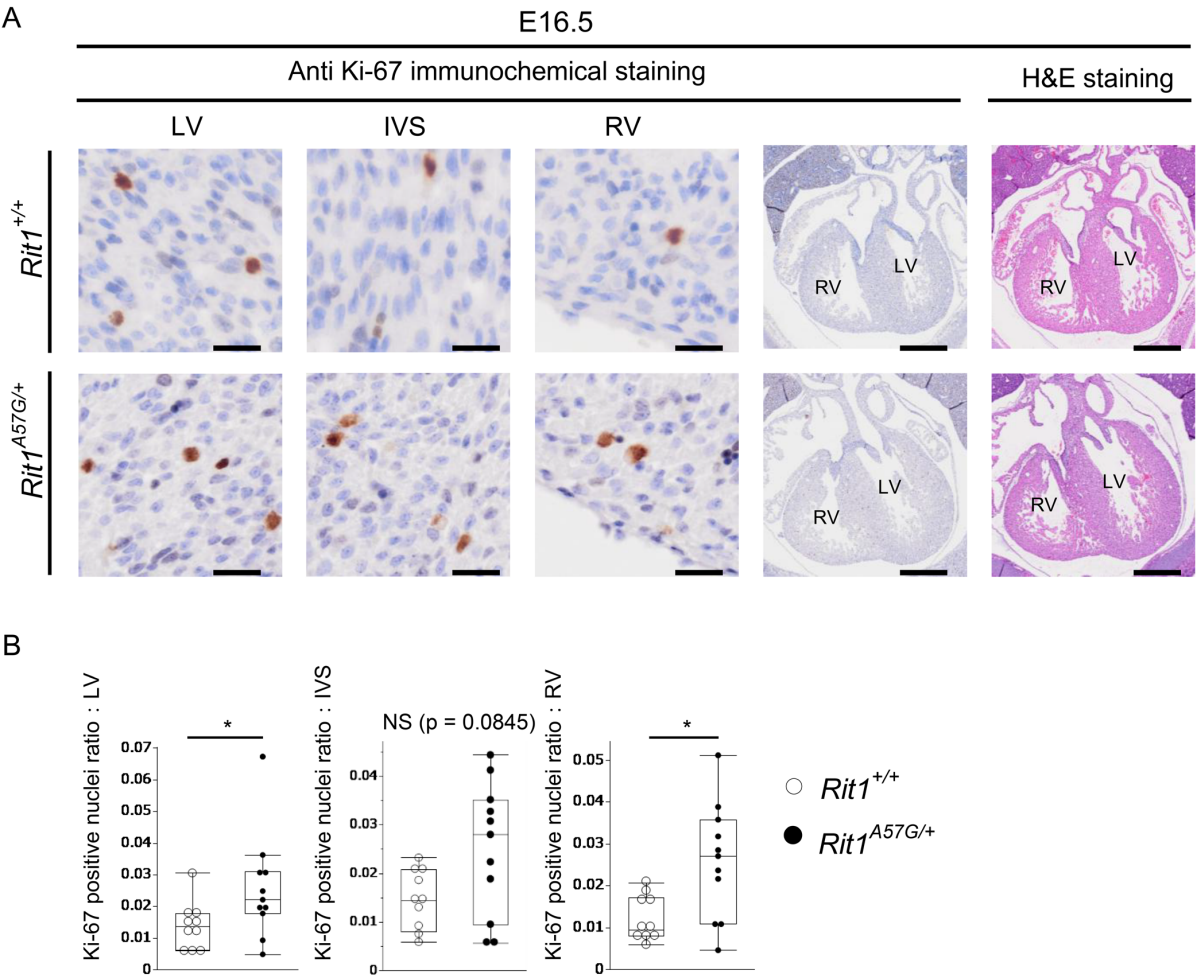
Rit1^A57G/+^ embryos showed increased cell proliferation at E16.5. (A) Representative images of heart sections stained with the anti‐Ki‐67 antibody at E16.5. The upper panels show Rit1^+/+^ embryos, and the lower panels show Rit1^A57G/+^ embryos. High magnification images indicate the LV wall, IVS, and RV wall of the heart, and low magnification images show a four‐chamber view of the heart with anti‐Ki‐67 immunochemical staining and hematoxylin–eosin staining. LV, left ventricle; IVS, interventricular septum; RV, right ventricle; Scale bars, 500 μm (low‐magnification) and 25 μm (high‐magnification). (B) The Ki‐67 positive nuclei ratio was evaluated and compared between the Rit1^+/+^ and Rit1^A57G/+^ embryos. Data are presented as median/IQR, Rit1^+/+^ *n* = 10, Rit1^A57G/+^ *n* = 11, *p < 0.05, NS: not significant (Wilcoxon signed‐rank sum test).

**FIGURE 4:**
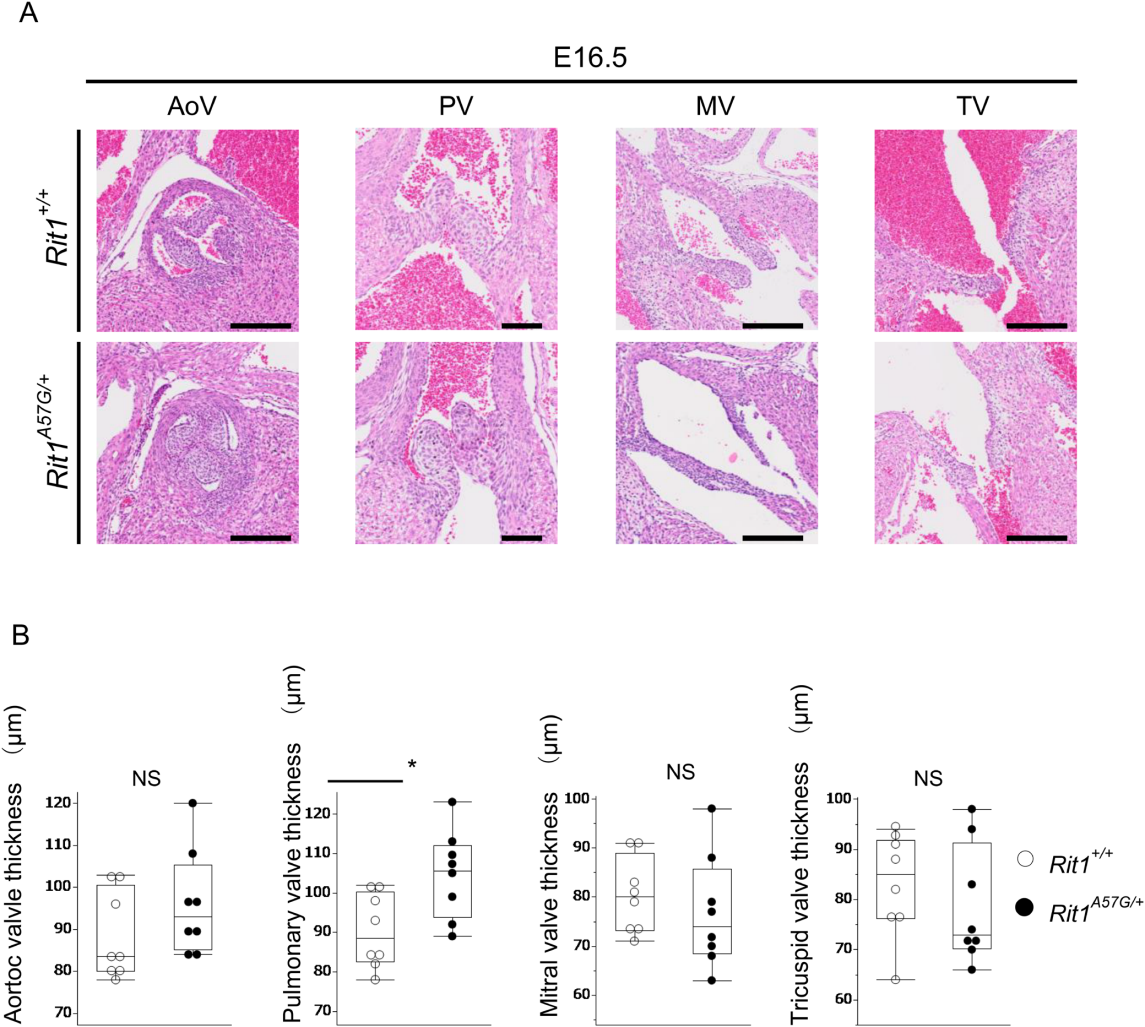
Rit1^A57G/+^ embryos exhibited pulmonary valve stenosis at E16.5. (A) Representative images of cardiac valves stained with hematoxylin and eosin at E16.5. Upper panels show Rit1^+/+^ embryos, and lower panels show Rit1^A57G/+^ embryos. The measured PV thicknesses are shown in the panels. AoV: aortic valve; PV: pulmonary valve; MV: Mitral valve; TV: Tricuspid valve; scale bars: 200 μm. (B) Cardiac valve thickness was measured and compared between Rit1^+/+^ and Rit1^A57G/+^ embryos. Data are presented as median/IQR, *n* = 8, *p < 0.05, NS, not significant (Wilcoxon signed‐rank sum test).

Patients with NS, particularly those with *RIT1* variants, exhibit HCM and pulmonary valve stenosis (PS) (Kouz et al. 2016; Zha et al. 2022). To investigate cardiovascular abnormalities in *Rit1* ^A57G/+^ embryos, we performed H&E staining at E14.5 and E16.5. The thicknesses of the left ventricular (LV) wall, intraventricular septum (IVS), and right ventricular (RV) wall were measured. At E14.5, the LV, IVS, and RV wall thicknesses (*n* = 8 in each group) and cell proliferation (wild‐type *n* = 7, *Rit1* ^A57G/+^ *n* = 8) were not significantly different between the *Rit1* ^A57G/+^ and *Rit1* ^+/+^ embryos (Figures S2 and S3). In contrast, at E16.5, the LV wall and IVS were thicker in the *Rit1* ^A57G/+^ embryos than in the *Rit1* ^+/+^ embryos (wild‐type *n* = 23, *Rit1* ^A57G/+^ *n* = 24). The RV wall thickness did not significantly increase in *Rit1* ^A57G/+^ embryos at E16.5 (Figure 2A,B). We hypothesized that the development of LV wall and IVS thickness might be due to increased proliferation. To test this hypothesis, we evaluated cell proliferation in embryonic hearts by Ki‐67 immunostaining. In *Rit1* ^A57G/+^ embryos, the Ki‐67 positive nuclei ratio was increased in the LV wall, but not in the IVS (wild‐type *n* = 10, *Rit1* ^A57G/+^ *n* = 11) (Figure 3A,B). Interestingly, the Ki‐67 positive nuclei ratio was increased in the RV wall (Figure 3A,B). Subsequently, we evaluated the cardiomyocyte size by counting the cardiomyocytes in a defined area of the LV. The average myocyte size in the LV of the *Rit1* ^A57G/+^ and *Rit1* ^+/+^ embryos was not significantly different at E16.5 (wild‐type *n* = 6, *Rit1* ^A57G/+^ *n* = 12) (Figure S4). Next, we evaluated cardiac valve thickness in embryos at E16.5. Consequently, only the pulmonary valve in *Rit1* ^A57G/+^ embryos exhibited increased thickness compared with that in *Rit1* ^+/+^ mice (*n* = 8 in each group) (Figure 4A,B). Congenital heart defects were also observed. Four cases of atrial septal defects (ASD) and one case of ventricular septal defect were identified in ten *Rit1* ^A57G/+^ embryos, whereas one case of ASD was observed in 11 *Rit1* ^+/+^ embryos at E16.5. These results suggest that *Rit1* ^A57G/+^ embryos at E16.5 have HCM and PS, as reported in human cases (Kouz et al. 2016; Zha et al. 2022).

### 3.4 Effect of MEK1/2 Inhibitor Treatment on Cardiac Phenotypes in *Rit1* ^A57G^ ^/+^ Mice

Previous studies have shown that MEK1/2 inhibitors improve cardiac phenotypes (Abe et al. 2024; Cuevas‐Navarro et al. 2023) and rescue embryonic lethality (Chen et al. 2010; Hernández‐Porras et al. 2014; Inoue et al. 2014) in RASopathy model mice. To assess the effect of MEK1/2 inhibitor treatment on cardiac phenotypes in *Rit1* ^A57G/+^ embryos, we treated pregnant mice with the MEK1/2 inhibitor PD0325901 and evaluated their embryos at E16.5. We administered 1 mg/kg PD0325901 to pregnant mice on gestational day 12.5 and 14.5, because fetal myocardial wall development accelerates after gestational day 12.5 (Petersen et al. 2023). Treatment with PD0325901 did not affect the ratio of *Rit1* ^A57G/+^ to *Rit1* ^+/+^ embryos compared to untreated embryos. The LV wall and IVS thickness decreased in PD0325901‐treated *Rit1* ^A57G/+^ embryos, but not in the RV wall (PD0325901‐untreated *n* = 24, PD0325901‐treated *n* = 12) (Table 2). Ki‐67 immunostaining revealed that cell proliferation in the LV wall was decreased in PD0325901‐treated *Rit1* ^A57G/+^ embryos (PD0325901‐untreated *n* = 11, PD0325901‐treated *n* = 8) (Table 3). In contrast, cardiac valve thickness did not show clear changes following MEK1/2 inhibitor administration (pulmonary valve thickness (median (interquartile range); PD0325901‐untreated (*n* = 8), 106（96‐112), PD0325901‐treated (*n* = 7), 98 (83‐115)). Taken together, MEK1/2 inhibitor administration influenced LV myocardial wall development, but did not affect cardiac valve development. These results suggest that treatment with a MEK1/2 inhibitor prevents cardiac hypertrophy.

**TABLE 2:** Thickness of cardiac walls in *Rit1* ^A57G/+^ embryos without treatment and PD0325901‐treated *Rit1* ^A57G/+^ embryos at E16.5.

	*n*	LV (μm)	IVS (μm)	RV (μm)
*Rit1* ^A57G/+^ embryos^a^				
Median (interquartile range)	24	200 (189–220)	298 (279–350)	126 (106–153)
PD0325901‐treated *Rit1* ^A57G/+^ embryos				
Median (interquartile range)	12	180 (102–219)	260 (232–311)	113 (101–145)
*p* value		0.044	0.030	0.32

^a^ The data of *Rit1* ^A57G/+^ embryos were used from those shown in Figure 2B.

**TABLE 3:** The ratio of Ki‐67 positive nuclei in and *Rit1* ^A57G/+^ embryos without treatment and PD0325901‐treated *Rit1* ^A57G/+^ embryos at E16.5.

	*n*	LV	IVS	RV
*Rit1* ^A57G/+^ embryos^a^				
Median (interquartile range)	11	0.0220 (0.0178–0.0310)	0.0280 (0.0095–0.0352)	0.0270 (0.0109–0.0357)
PD0325901‐treated *Rit1* ^A57G/+^ embryos				
Median (interquartile range)	8	0.0133 (0.0105–0.0177)	0.0194 (0.0104–0.0210)	0.0181 (0.0108–0.0222)
*p* value		0.035	0.17	0.091

^a^ The data of *Rit1* ^A57G/+^ embryos were used from those shown in Figure 3B.

## 4 Discussion

In this study, we demonstrated LV wall and IVS hypertrophy and pulmonary valve hyperplasia in *Rit1* ^A57G/+^ embryos at E16.5. Cardiac hypertrophy was caused by progressive cell proliferation without cardiomyocyte hypertrophy. These findings were not observed at E14.5. Neck‐to‐head lymphatic vessels were dilated in *Rit1* ^A57G/+^ embryos at E16.5. Prenatal administration of a MEK1/2 inhibitor to *Rit1* ^*A57G/+*^ embryos prevented cardiac hypertrophy.

The clinical diagnosis of HCM is based on a hypertrophied, non‐dilated left ventricle. Epidemiological studies have shown that the disease prevalence in the general population is 1 case per 200–500 individuals (Maron 2018). It is estimated that up to 60% of cases are attributable to mutations in genes encoding sarcomeric proteins, including beta‐myosin heavy chain, myosin‐binding protein C, troponin I, and troponin T (Medical Masterclass and Firth 2019). RASopathies are the most prevalent congenital malformation syndrome causing HCM (Sebastian et al. 2023). The incidence of HCM in patients with NS is 10%–48% (Ilic et al. 2024; Roberts et al. 2013; Sun et al. 2022), with early onset and frequent complications of congestive heart failure, rendering it a more severe condition (Chen et al. 2019; Wilkinson et al. 2012). Common histological findings in HCM include myocyte hypertrophy, disorganized myocytes, normal myocardial architecture (myocardial disarray), and interstitial fibrosis (Ishibashi‐Ueda et al. 2017; Marian and Braunwald 2017; Shirani et al. 2000). Histological analyses have demonstrated that ventricular hypertrophy associated with NS resembles nonsyndromic HCM, particularly in terms of the degree of myocardial disarray (Burch et al. 1992). Furthermore, patients with NS exhibit a higher degree of endocardial fibrosis but a lower degree of myocyte hypertrophy (Poterucha et al. 2015). In mouse models, a high frequency of Ki‐67 positive nuclei was observed in the hearts of *Rit1* ^A57G/+^ and *Kras* ^V14I^ adult mice (Hernández‐Porras et al. 2014; Takahara et al. 2019). We have shown that the hearts of *Rit1* ^A57G/+^ mice at 12 and 26 weeks exhibited increased fibrosis, but no cardiomyocyte hypertrophy (Takahara et al. 2019). In the present study, cardiomyocyte hypertrophy was not observed in the hypertrophied LV walls of *Rit1* ^A57G/+^ embryos at E16.5, whereas the number of cardiomyocytes was increased in the LV walls (Figure S4). Collectively, our results indicate that the HCM in *Rit1* ^A57G/+^ embryos as well as that in adult *Rit1* ^A57G/+^ mice^18^ exhibits an increase in cell number but not in cell hypertrophy.

Recent clinical studies have reported the administration of the MEK1/2 inhibitor trametinib in patients with RASopathies associated with HCM (Andelfinger et al. 2019; Hribernik et al. 2023; Kiamanesh et al. 2024; Leegaard et al. 2022; Meisner et al. 2021; Mussa et al. 2021; Nakano et al. 2022). Most patients exhibited improvement in HCM symptoms, with the exception of two fatalities (Mussa et al. 2021; Nakano et al. 2022). There were no side effects other than diarrhea and skin rash (Hribernik et al. 2023; Leegaard et al. 2022; Meisner et al. 2021). Moreover, there have been no reports on the administration of pathway‐specific inhibitors to human fetuses. In mouse models, prenatal treatment with a MEK1/2 inhibitor decreased ERK1/2 activation and rescued embryonic lethality in *Sos1* ^E846K/E846K^ and *Kras* ^V14I/+^ mice, respectively (Chen et al. 2010; Hernández‐Porras et al. 2014). We have also reported that fetal administration of a MEK1/2 inhibitor to *Braf* ^Q241R/+^ mice partially rescued embryonic lethality and prevented craniofacial abnormalities and edema (Inoue et al. 2014). In this study, the MEK1/2 inhibitor was effective in improving the hypertrophy of the LV wall and IVS (Tables 2 and 3), but not the thickness of the cardiac valve. These results suggest that the MEK/ERK activation would be one of the pathogenetic mechanisms of cardiac hypertrophy in *Rit1* ^A57G/+^ embryos. Further studies will be necessary to understand the detailed mechanisms of cardiac hypertrophy and the development of valves in *Rit1* ^A57G/+^ embryos.

Another important complication of patients with NS is lymphatic abnormalities, including congenital lymphedema, chylothorax, or ascites in the neonatal period and increased nuchal translucency, distended jugular lymphatic sacs, or pleural effusion in the prenatal period (Zenker 2022). Of patients with NS, lymphatic abnormalities are frequently observed in patients with NS harboring *SOS2* and *RIT1* mutations (Lissewski et al. 2021; Zha et al. 2022). Our review of 14 patients with NS exhibiting *RIT1* mutations revealed that all patients presented with lymphatic disorders during the fetal and neonatal periods (Yaoita et al. 2016). A previous study showed that half of the *Rit1* ^A57G/+^ mice died within two days after birth (Takahara et al. 2019). In the current study, lymphatic vessel dilatation was evident in *Rit1* ^A57G/+^ embryos at E16.5, but no other fetal lymphatic disorders such as pleural effusion or ascites were observed. One of the two embryos exhibiting systemic edema in gross appearance at E16.5 also showed nuchal edema in the transverse section upon H&E staining (Figure 1A). It is possible that lymphatic abnormalities may be one of the reasons for the prenatal or postnatal mortality in the *Rit1* ^A57G/+^ mice.

In summary, we demonstrated that *Rit1* ^A57G/+^ embryos exhibit LV wall and IVS hypertrophy without cardiomyocyte hypertrophy and present with pulmonary valve hyperplasia. These abnormal phenotypes were caused by increased cell proliferation. Furthermore, the MEK1/2 inhibitor PD0325901 prevented cardiac hypertrophy in *Rit1* ^A57G/+^ embryos. These findings indicate that the *RIT1* mutation causes cardiovascular abnormalities in the fetal period, and that the activation of MEK/ERK is the potential pathogenesis of cardiac hypertrophy.

## Author Contributions

D.S., T.A., and Y.A. designed experiments. D.S. performed experiments. T.N. supported data analysis. Y.A. obtained the funding for this study. D.S. wrote the draft. T.A., T.N., A.K., and Y.A. revised the manuscript.

## Funding

This work was supported by Japan Society for the Promotion of Science (JP23K27563 and JP21K19436), Japan Agency for Medical Research and Development (JP23ek0109618).

## Conflicts of Interest

The authors declare no conflicts of interest.

## Supporting information


**Data S1:** mgg370167‐sup‐0001‐DataS1.pdf.

## Data Availability

All the data and materials analyzed in this study are available from the corresponding author upon reasonable request.
