# Supplementary material for: Phenotypic Analysis of Embryos in a Noonan Syndrome Model Mouse With the Rit1 A57G Mutation
Source: Mol Genet Genomic Med. 2025 Dec 13;13(12):e70167. doi: 10.1002/mgg3.70167 (PMC12701617; doi:10.1002/mgg3.70167)
Supplement: Supplementary file 1 — Data S1: mgg370167‐sup‐0001‐DataS1.pdf. [file MGG3-13-e70167-s001.pdf]

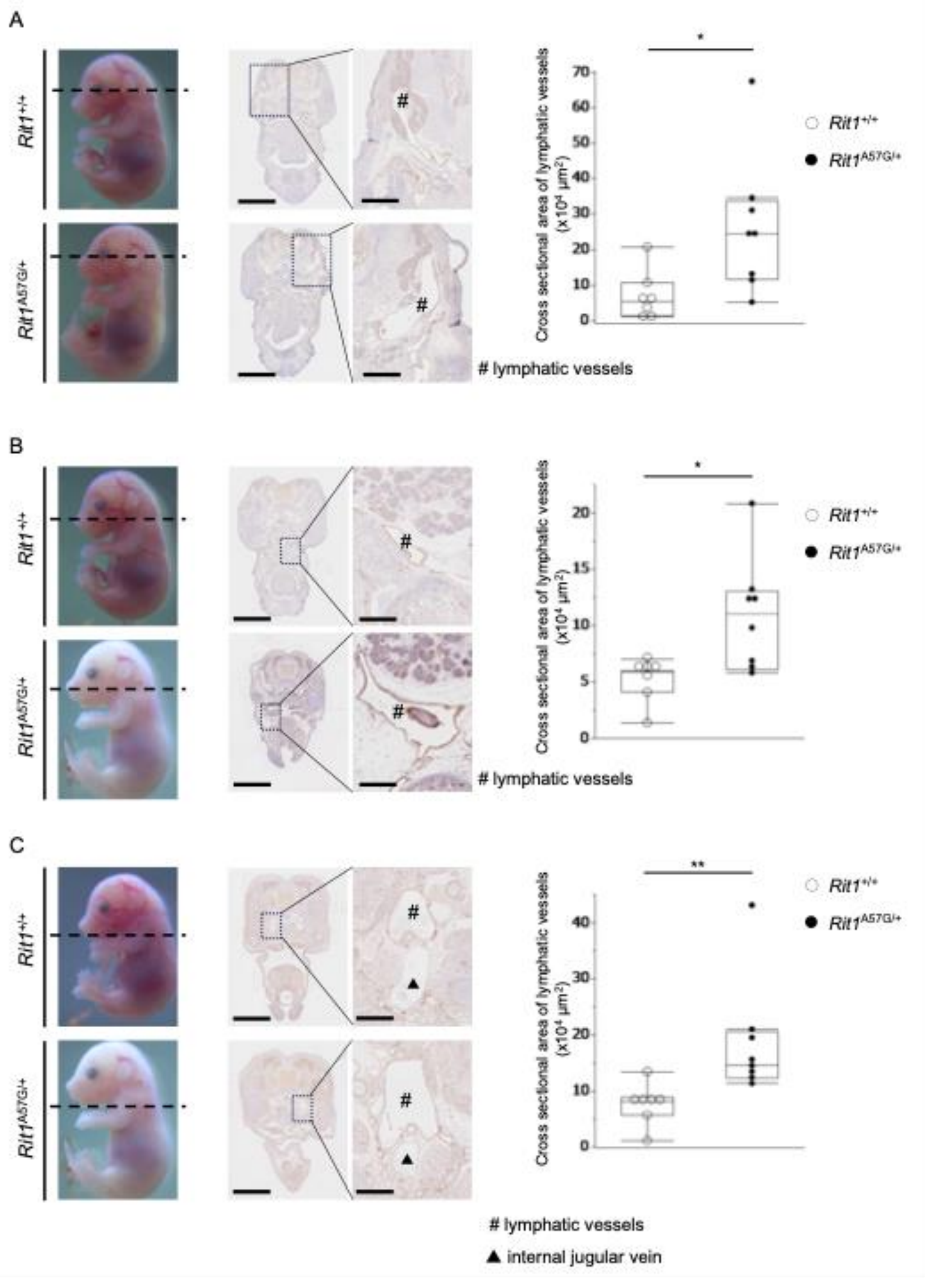

Supplementary Fig. 1. Lymphatic vessel abnormalities in *Rit1*$^{\mathrm{A57G/+}}$ embryos at E16.5.

Lymphatic vessels were measured and compared between *Rit1*$^{+/+}$ and *Rit1*$^{A57G/+}$ embryos. (A), (B), and (C) indicate lymphatic vessels located around the temporal region, lymphatic vessels located around mandible, and the cervical lymphatic sac, respectively. Representative images of the gross appearance of an embryo at E16.5, low- and high-magnification images of anti-VEGFR3 immunochemical staining, and comparison of lymphatic vessels. The dotted line indicates the height of the section. Scale bars, 2 mm (low-magnification), 500 µm (high-magnification in A), and 200 µm (high-magnification in B and C). Data are presented as median/IQR, *Rit1*$^{+/+}$ (n = 7), *Rit1*$^{A57G/+}$ (n = 8), * $p < 0.05$, ** $p < 0.01$ (Wilcoxon signed-rank sum test).

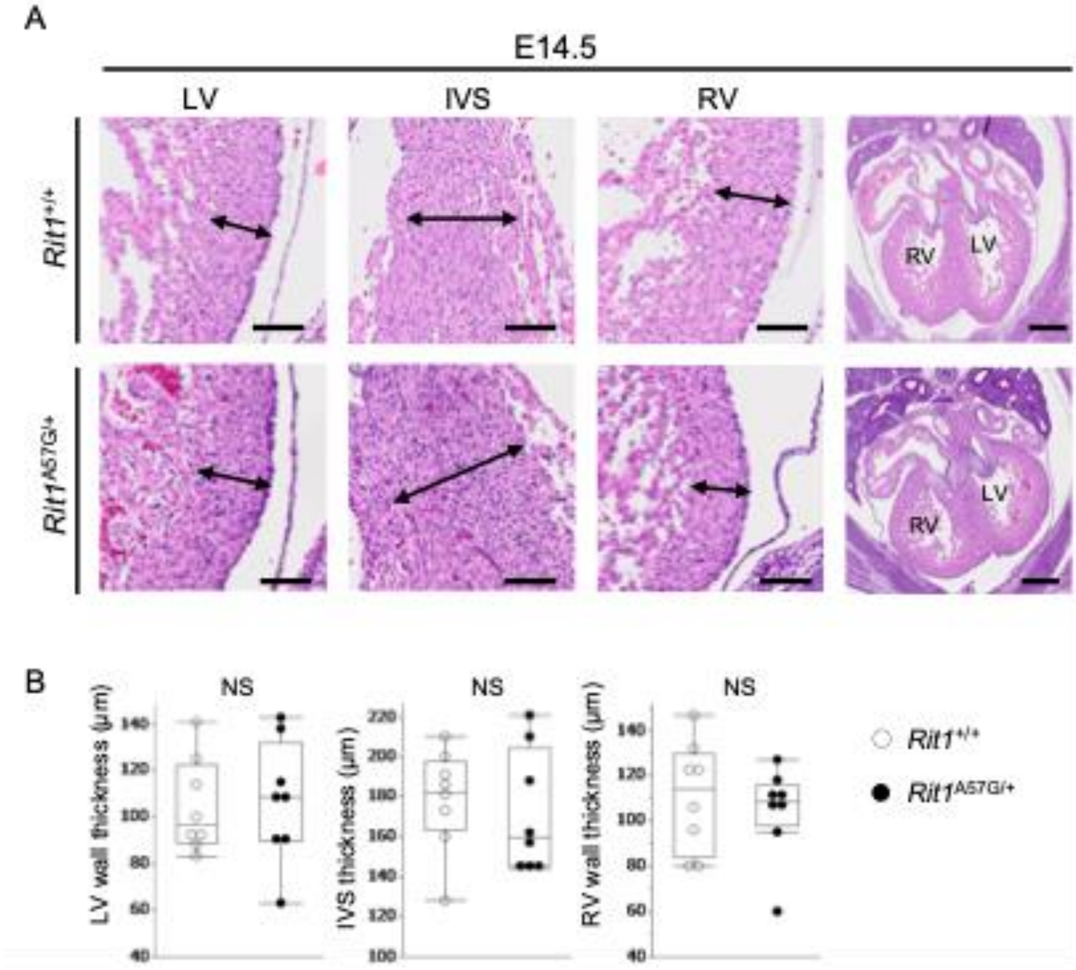

Supplementary Fig. 2. ***Rit1*$^{\mathbf{A57G/+}}$ embryos did not exhibit ventricular hypertrophy at E14.5.** (A) Representative images of heart sections stained with hematoxylin and eosin at E14.5. The upper panels show *Rit1*$^{+/+}$ embryos, and the lower panels show *Rit1*$^{A57G/+}$ embryos. High-magnification images indicate LV wall, IVS, and RV wall of the heart, and low-magnification images show a four-chamber view of the heart. The measured wall thickness is shown for each high-magnification image. LV, left ventricle; IVS, interventricular septum; RV, right ventricle; Scale bars, 500 µm (low-magnification) and 100 µm (high-magnification). (B) Ventricular wall thickness was measured and compared between *Rit1*$^{+/+}$

and *Rit1*$^{A57G/+}$ embryos. Data are presented as median/IQR, n = 8, NS, not significant (Wilcoxon signed-rank sum test).

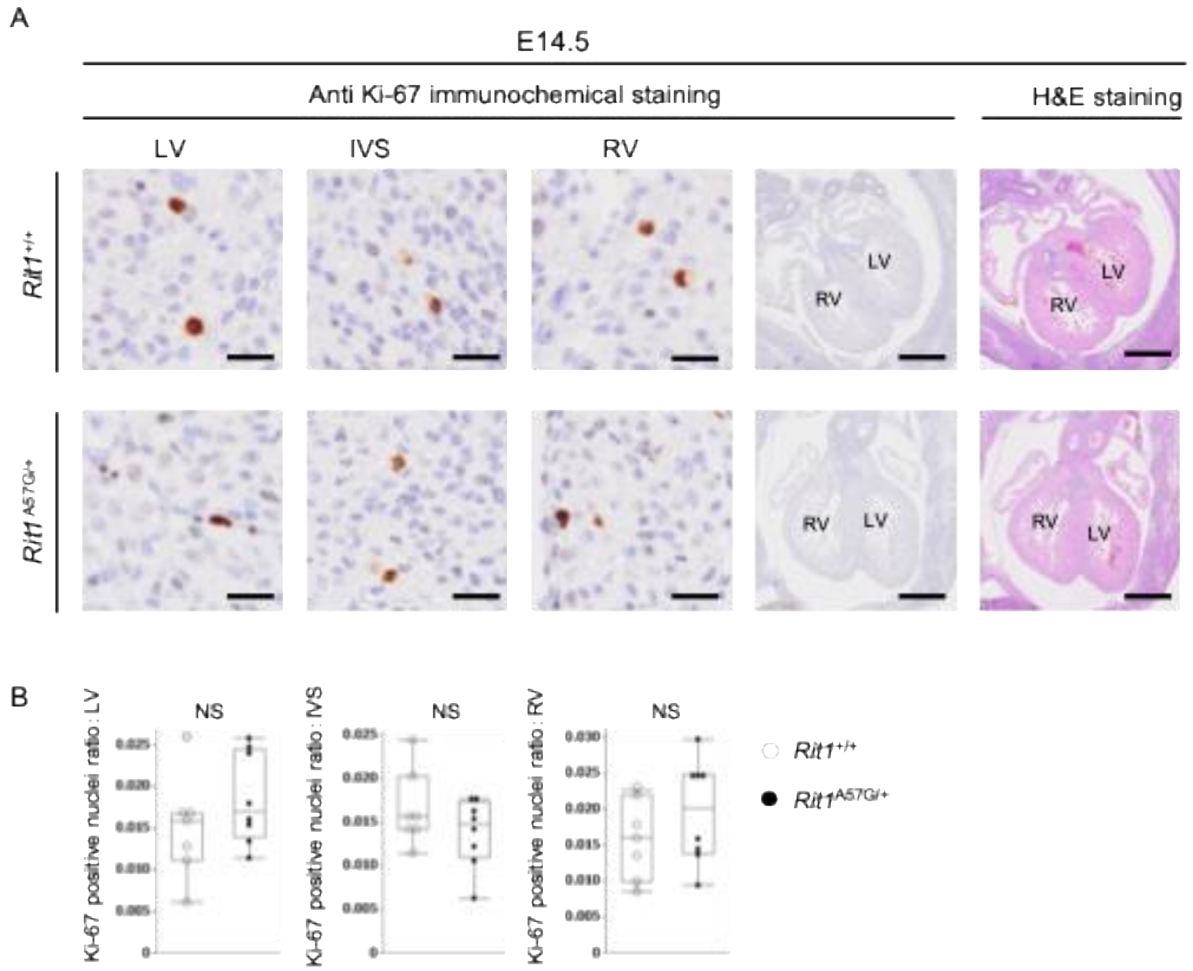

Supplementary Fig. 3. ***Rit1*$^{\mathbf{A57G/+}}$ embryos did not exhibit increased cell proliferation at E14.5.** (A) Representative images of heart sections stained with the anti-Ki-67 antibody at E14.5. The upper panels show *Rit1*$^{+/+}$ embryos, and the lower panels show *Rit1*$^{A57G/+}$ embryos. High-magnification images indicate the LV wall, IVS, and RV wall of the heart, and low-magnification images show a four-chamber view of the heart with anti-Ki-67 immunochemical and hematoxylin and eosin staining. LV, left ventricle; IVS, interventricular septum; RV, right ventricle; Scale bars, 500 µm (low-magnification) and 25 µm (high-magnification). (B) The Ki-67 positive nuclei ratio was evaluated and compared between

Rit1$^{+/+}$ and *Rit1*$^{A57G/+}$ embryos. Data are presented as median/IQR, *Rit1*$^{+/+}$ (n = 7), *Rit1*$^{A57G/+}$ (n = 8), NS, not significant (Wilcoxon signed-rank sum test).

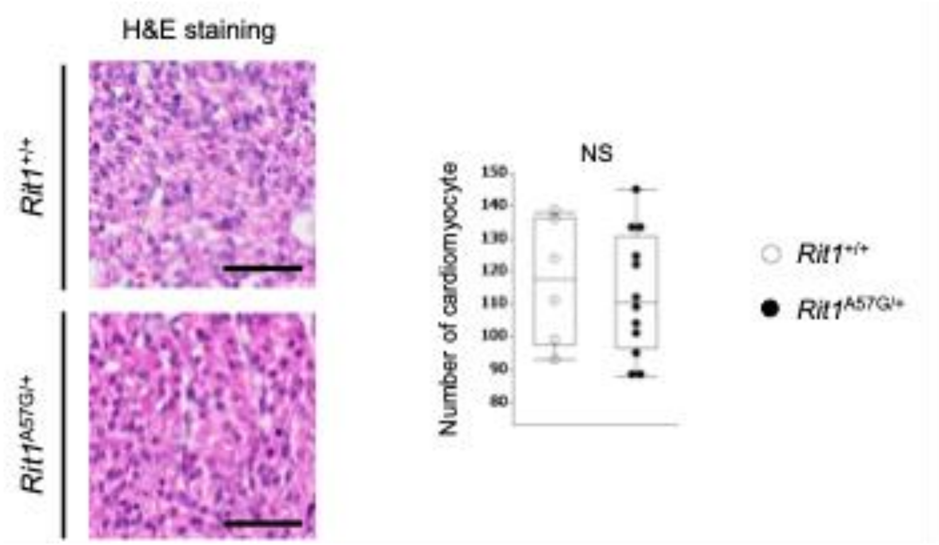

Supplementary Fig. 4. The average size of myocytes in the LV was not significantly different at E16.5. Representative images of heart sections from a range of 0.02mm^2 stained with hematoxylin-eosin at E16.5. Upper panels are *Rit1*$^{+/+}$ embryos, and lower panels are *Rit1*$^{A57G/+}$ embryos. The number of cardiomyocytes were measured and compared between *Rit1*$^{+/+}$ and *Rit1*$^{A57G/+}$ embryos. Data are presented as median/IQR, *Rit1*$^{+/+}$ (n = 6), *Rit1*$^{A57G/+}$ (n = 12), NS: not significant (Wilcoxon signed-rank sum test).
